# Clinicopathological and genotypic characteristics of colorectal cancer patients carrying a germline MUTYH mutation

**DOI:** 10.1590/1806-9282.20251212

**Published:** 2026-04-20

**Authors:** Ismail Guzelis, Roya Gasimli, Aslı Subaşıoğlu, Aysegul Sari

**Affiliations:** 1 Izmir Buca Seyfi Demirsoy Training and Research Hospital, Department of Pathology – İzmir, Turkiye.; 2 Dokuz Eylul University, Institute of Health Sciences, Department of Molecular Pathology – Izmir, Turkiye.; 3 Ege University, Department of Medical Biology – İzmir, Turkiye.; 4 Izmir Katip Celebi University, Ataturk Training and Research Hospital, Department of Medical Genetics – İzmir, Turkiye.; 5 Izmir Katip Celebi University, Ataturk Training and Research Hospital, Department of Pathology – İzmir, Turkiye.

**Keywords:** Colorectal cancer, Mismatch repair deficiency, MUTYH, Polyposis

## Abstract

**OBJECTIVE:**

Approximately 5–10% of the cases with colorectal cancers have a hereditary cancer syndrome. MUTYH is a DNA base excision repair gene, and its mutation can induce the development of polyposis and colorectal cancer. Additionally, MUTYH repair gene may interact with the DNA mismatch repair system. The aim of this study was to investigate the clinicopathological features of colorectal cancer cases carrying germline MUTYH mutations.

**METHODS:**

Among patients genetically tested using large hereditary cancer panels, data of those carrying germline MUTYH mutations were retrieved from the archive files. Then, the patients who had colorectal cancer were included in the study.

**RESULTS:**

Ten male and three female colorectal cancer patients with pathogenic (n=10) and variant of uncertain significance MUTYH mutations (n=3) were included in the study. While seven cases had homozygous MUTYH mutations, six patients carried heterozygous MUTYH mutations. The patients had c.800C>T p.P267L (n=4), c.1353_1355delGGA p.E452del (n=4), c.1087C>T p.Q363 (n=1), c.631G>A p.V211I (n=1), c.180A>G p.R60R (n=1), c.509G>A p.G170E (n=1) and c.650G>A p.R217H (n=1) mutations. The ascending colon was the most common site of involvement. Six cases with homozygous and one case with double heterozygous mutations had polyposis. Cases with homozygous (n=1) and heterozygous (n=1) MUTYH mutations were found to be MSH2-deficient. In both MSH2-deficient cases, the tumors were located in the ascending colon, and the case with a homozygous mutation had >100 polyps.

**CONCLUSION:**

Given that the MUTYH mutation is rarely seen in cases of colorectal cancers, we believe that our findings may contribute to identifying potential clinical and therapeutic implications for individuals with this mutation.

## INTRODUCTION

Colorectal cancer (CRC) is the third most common cancer in the world^1^. As screening protocols develop, it has been shown that 60% of CRCs can be prevented. Approximately 5–10% of cases with colorectal cancer have a hereditary cancer syndrome. Hereditary CRC is classified as polyposis and nonpolyposis CRC^2^. Hereditary nonpolyposis CRC (HNPCC) syndrome, also termed Lynch syndrome, is responsible for 80% of the cases of hereditary CRC^3^. Mismatch repair genes MSH-2, MSH-6, MLH-1, and PMS-2, are the defective genes in the Lynch syndrome^4^. All newly diagnosed CRC patients go through screening for Lynch syndrome. Several criteria should exist to establish the diagnosis of adenomatous polyposis. The most important criterion is the presence of ≥20 adenomatous polyps^5^. Familial adenomatous polyposis (FAP) is a well-defined prototype of polyposis-related hereditary CRC. FAP is characterized by more than 100 polyps in the colon and extracolonic locations. If not prevented, there is a 100% probability of developing CRC by the age of 45 in FAP patients^6^. However, FAP develops in only 0.5–1% of cases with CRC^7^. The recommended surveillance protocols for FAP patients are being implemented at the age of 10–15 years. Total colectomy is the ideal treatment when the disease cannot be cured with endoscopic interventions^8^.

MUTYH is a DNA base excision repair gene^9^. The clinical features of MUTYH-associated polyposis (MAP) are ­similar to those of FAP. MAP differs from FAP with its late onset and the presence of polyposis, consisting typically of 10–100 ­polyps^10^. Due to its onset in advanced age, cases with MAP are ­recommended to be screened from the age of 25 years. If the adenoma burden cannot be managed endoscopically, inevitably, total colectomy options should be considered^8^. While homozygous germline mutations in MUTYH are associated with polyposis, heterozygous mutations are linked to an increased risk of colorectal, gastric, and breast cancers^11^. There are limited studies on MUTYH-mutated CRC and its genotypic features^12-14^. A few studies have demonstrated mismatch repair (MMR)-deficiency in MUTYH-mutated patients^15-17^. To the best of our knowledge, no studies have investigated the clinicopathological features of MUTYH-mutated colorectal cancer in the Turkish population, and there are only limited studies on this topic overall^15,16,18^. This study aimed to illustrate the clinicopathological attributes and genotyping of colorectal cancer patients carrying a germline MUTYH mutation in the Turkish population.

## METHODS

Patients were referred to the Medical Genetics Department of Izmir Katip Celebi University Ataturk Training and Research Hospital for genetic screening. Peripheral blood samples were collected from these patients. Library preparation was performed using germline QIAseq®Targeted DNA Custom panel kits, which included 61 genes associated with hereditary cancer and are designed for Illumina sequencing systems. Variants were classified based on their pathogenicity as variants of uncertain significance (VUS), likely pathogenic, or pathogenic (P), and their correlation with the patients’ clinical phenotypes was examined. Patients who were found to carry MUTYH mutations were retrieved from the archive data. The clinical data of the patients were subjected to a comprehensive examination within the hospital database. The cases of colorectal cancer were identified and included in the study. The clinicopathological features of these cases were investigated in the database of the pathology department. The study approval was obtained from the institutional review board of Izmir Kâtip Celebi University medical faculty.

### Statistical analysis

The data were statistically evaluated with the R-based statistical program Jamovi version 2.3. Descriptive statistics were used for categorical and continuous variables.

## RESULTS

A total of 13 colorectal cancer patients including 10 male, and 3 female cases with pathogenic, likely pathogenic or MUTYH mutation VUS were included in the study. Ten cases had pathogenic MUTYH mutations, and three had VUS MUTYH mutations. The median age of the subjects was 56 years. The patients had c.800C>T p.P267L (n=4), c.1353_1355delGGA p.E452del (n=4), c.1087C>T p.Q363 (n=1), c.180 A>G p.R60R (n=1), c.509 G>A p.G170E (n=1), c.631G>A p.V211I (n=1), and c.650 G>A p.R217H (n=1) mutations. While seven cases had homozygous MUTYH mutations, six carried heterozygous MUTYH mutations. One of the heterozygous cases carried c.1353_1355delGGA p.E452del and c.452 A>G p.Y151C mutations. Total colectomy was performed on nine (69.2%) patients. Four cases had synchronous adenocarcinomas in distinct colonic locations (30.8%). The ascending colon was the most commonly affected site with 7 (53.8%) cases. Pathological T stages in respective number of cases were as follows: pT0 (n=1), pT2 (n=1), pT3 (n=8), and pT4 (n=3). While some cases had moderately (n=8), poorly (n=4), and well-differentiated (n=1) adenocarcinomas. Lymph node metastasis was detected in six (46.2%) cases. Six cases with homozygous and one case with double heterozygous mutation had polyposis. A minimum of 36 and a maximum of 130 polyps were observed in cases with polyposis. The clinicopathological and genotypical features of the cases are summarized in Table 1.

**Table 1:** Clinicopathological and genotypical characteristics of the cases.

Patients (N=13)		N (%)
MUTYH mutations, n (%)		
	c.800 C>T p.P267L	4 (30.8)
	c.1353_1355delGGA p.E452del	4 (30.8)
	c.1087 C>T p.Q363	1 (7.7)
	c.631 G>A p.V211I	1 (7.7)
	c.650 G>A p.R217H	1 (7.7)
	c.180 A>G p.R60R	1 (7.7)
	c.509 G>A p.G170E	1 (7.7)
	Homozygous MUTYH mutations, n (%)	7 (53.8)
	Multiple carcinomas, n (%)	4 (30.8)
	Median tumor diameter (cm)	4.3
pT, n (%)		
	0	1 (7.7)
	2	1 (7.7)
	3	8 (61.5)
	4	3 (23.1)
Histological grade, n (%)		
	1	1 (7.7)
	2	8 (61.5)
	3	4 (30.8)
pN, n (%)		
	0	7 (53.8)
	1	2 (15.4)
	2	4 (30.8)
	Polyposis, n (%)	7 (53.8)
	Median number of polyps	41.3
Results of MMR protein analyses		
	NA	1 (7.7%)
	MMR-intact	10 (76.9%)
	MMR-deficient	2 (15.4%)

N is the number of non-missing values. MMR: mismatch repair; NA: not available.

### Cases with homozygous MUTYH mutations

Seven cases with homozygous MUTYH mutations had c.800 C>T p.P267L (n=3), c.1353_1355delGGA p.E452del (n=2), c.1087 C>T p.Q363 (n=1), and c.650 G>A p.R217H (n=1) mutations. Six cases presented with polyposis. The only case without polyposis had a c.650 G>A p.R217H mutation. This case also had undergone mastectomy for invasive breast ­cancer-no special type.

### Mismatch repair protein analyses

When MMR immunohistochemistry was applied to 12 cases, two cases were found to be MSH2-deficient, while the remaining cases were intact. Both MSH2-deficient cases had tumors located in the ascending colon and they underwent total colectomy. One of the MMR-deficient cases exhibited a heterozygous c.631 G>A p.V211I mutation variant of uncertain significance. There were synchronous CRCs without polyposis. The tumor in the ascending colon was a moderately differentiated adenocarcinoma, whereas the one in the sigmoid colon was well differentiated. The pT3 tumor in the ascending colon ­exhibited MSH2 deficiency, while the pT4 tumor in the sigmoid colon was MMR-intact. All excised lymph nodes were reactive.

The MMR-deficient case with a homozygous c.1353_1355delGGA p.E452del mutation had 105 polyps, along with a ­moderately differentiated unifocal adenocarcinoma in the ­ascending colon. The tumor was classified as pT3, and no lymph node metastasis was detected. The detailed clinicopathological and genotypical information for each case is presented in Table 2.

**Table 2:** The detailed clinicopathological and genetic information of each case.

Case number	Code/rs number/zygosity	Histological type and grade	Tumor size	pT	pN	Polyps, n	MMR status
1	c.800 C>T p.P267L/rs374950566/homozygous	Adenocarcinoma, G2	5 cm	pT3	pN 0	36	Intact
2	c.1087 C>T p.Q363/rs587783057/homozygous	Adenocarcinoma, G1	3 cm	pT0	pN 0	130	NA
3	c.452 A>G p.Y151C/rs34612342/heterozygous	Both Adenocarcinoma, G2	Transverse colon-2.8 cm	pT3	pN 0	60	Intact
	c.1353_1355delp.E452del/rs587778541/heterozygous		Rectum-3.2 cm	pT1			
4	c.631 G>A p.V211I/rs759295912/heterozygous	Ascending colon—Adenocarcinoma, G2	3.5 cm	pT3	pN 0	1	Loss
		Sigmoid colon—Adenocarcinoma, G1	3.2 cm	pT2			
5	c.1353_1355delGGA p.E452del/rs587778541/homozygous	Signet ring cell—G3	4.5 cm	pT4b	pN 2b	86	Intact
6	c.800 C>T p.P267L/rs374950566/homozygous	Ascending colon—Mucinous, G2	4.5 cm	pT2	pN 1b	40	Intact
		Transverse colon—Adenocarcinoma, G2	5 cm	pT3			
7	c.650 G>A p.R217H/rs140342925/homozygous	Adenocarcinoma, G3	6 cm	pT4b	pN 2a	0	Intact
8	c.800 C>T p.P267L/rs374950566/heterozygous	Adenocarcinoma, G2	2.5 cm	pT3	pN 0	3	Intact
9	c.1353_1355delGGA p.E452del/rs587778541/homozygous	Adenocarcinoma, G2	6 cm	pT3	pN 0	105	Loss
10	c.1353_1355delGGA p.E452del/rs587778541/heterozygous	Adenocarcinoma, G3	3 cm	pT4b	pN 2a	0	Intact
11	c.800C>T p.P267L/rs374950566/homozygous	Caecum—Adenocarcinoma, G2	6.5 cm	pT3	pN 2b	70	Intact
		Rectum—Adenocarcinoma, G3	2.8 cm	pT3			
12	c.180 A>G p.R60R/rs1553130310/heterozygous	Adenocarcinoma, G2	3.5 cm	pT3	pN 0	0	Intact
13	c.509 G>A p.G170E/rs1645156886/heterozygous	Adenocarcinoma, G2	6 cm	pT3	pN 1b	6	Intact

G: grade; MMR: mismatch repair protein analyses; NA: not available; rs number: reference SNP cluster ID.

### Colorectal cancer cases with c.800C>T p.P267L or c.1353_1355delGGA p.E452del mutations

None of the four cases with the c.800C>T p.P267L mutation showed loss of MMR proteins. Three of these mutations were homozygous and presented with polyposis. Four cases with c.1353_1355delGGA p.E452del mutations were detected. Two of these were homozygous mutations presenting with polyposis. One of the cases with a heterozygous c.1353_1355delGGA p.E452del mutation had a concomitant c.452 A>G p.Y151C mutation. Among the cases with heterozygous MUTYH mutations, this case with double heterozygosity was the only case that presented with polyposis.

### Cases with mutations that are variants of uncertain significance

Three cases were identified with a MUTYH mutation that is a variant of uncertain significance. All of these mutations were heterozygous. The case with a c.180 A>G p.R60R mutation had undergone low anterior resection. A pT3, MMR-intact CRC was identified in the rectum. The case with a c.509 G>A p.G170E mutation had undergone total colectomy. A tumor with a diameter of 6 cm was located in the descending colon. Lymph node metastasis was observed.

## DISCUSSION

Genetic counseling for adenomatous polyposis requires the identification of some criteria. Having ≥20 adenomas, family history of polyposis syndromes, and congenital hypertrophy of the retinal pigment epithelium are most frequently well-established criteria. While FAP is the most common hereditary cause of polyposis-associated CRC, MUTYH needs to be tested^8^. Symptomatic patients with a MUTYH mutation so-called MAP, can present with CRC at older ages^5,8^. Initially, it was hypothesized that MAP occurred due to the mutation of a few specific gene loci in MUTYH. As a result of advances in sequencing protocols and research on different ethnic groups, a number of MUTYH mutations have been identified^18-22^. The estimated lifetime risk of developing CRC in patients with MAP is approximately 80%^23^. Given that metachronous and possibly missed synchronous CRCs are rarely seen, patients who have undergone segmental colon resections should be monitored closely ^24^.

The c.800C>T p.P267L and c.1353_1355delGGA p.E452del MUTYH mutations were the most prevalent MUTYH mutations in our study. Seven cases with homozygous MUTYH mutations, also including three cases with c.800 C>T p.P267L, and two cases with c.1353_1355delGGA p.E452del mutations were observed. A comprehensive investigation conducted in Erzurum, Turkey revealed that the majority of the cases exhibiting either CRC or polyposis manifested the c.800C>T p.P267L variant in MUTYH mutation. In the same study, the second most common MUTYH mutation was identified as c.1353_1355delGGA p.E452del^12^. In a comprehensive investigation conducted at a single center in Ankara, Turkey, four distinct DNA alterations (c.536A>G p.T179C, c.545G>A p.A182H, c.884C>T p.P295L, and c.14734_1439delGGA p.G480del) were identified in five CRC patients with MUTYH mutations. The mutations identified in the study by Erdem et al. were not observed in our database^13^. A variation which had the same rs587783057 number as c.1087 C>T p.Q363 mutation was previously reported in a Turkish patient ^25^.

The MUTYH mutations described in our study and in ­studies on Turkish populations differ from the MUTYH mutation variants reported from other countries^12,13,15,18,24^. The G393D, G396D, and Y179C MUTYH mutations were the most common mutations cited in the scientific literature regarding the Spanish population^15,16^. In a multicenter study performed in Bonn, Cardiff and Leiden, Y179C and G396D were frequently observed MUTYH mutations^18^. In the Finnish population, Y165C and G382D were the most common MUTYH mutations^19^. Rare and singular variants of the MUTYH mutations were observed in Japanese populations^21,22^. In the United Kingdom, a population consisting mostly of Indian descent showed the G480T MUTYH mutation^24^. In the United States, p.T165C and p.G382A mutations were investigated^20^. In an Italian cohort, the two most common MUTYH pathogenetic variants were c.536A4G p.Tyr179Cys and c.1187G4A p.Gly396Asp^26^. In the Portuguese population, c.494A>G p.Y165C was the most frequently detected homozygous mutation^27^. A summary of common MUTYH mutations from different ethnicities is provided in Table 3.

**Table 3:** Summary of common MUTYH mutations across populations.

Population	Common MUTYH mutations
Spanish^15,16^	G393D, G396D, Y179C
German, Weltch, and Dutch^18^	G396D, Y179C
Finnish^19^	G382D, Y165C
American^20^	G382A, T165C
Indian^24^	G480T
Italian^26^	G396A, T179C
Portuguese^27^	Y165C
Turkish^12^ (including our cohort)	P267L, E452del

Six out of seven cases with homozygous MUTYH mutations were associated with polyposis. Homozygous MUTYH mutations are known to be the underlying factor for the development of MAP^11^. Only a single case that carried double heterozygous MUTYH mutations presented with polyposis. In the literature, a case with a double heterozygosity in MUTYH has been reported. Similarly to our case, the reported case presented with a CRC and polyposis^28^. Patel et al. previously reported that the type of colectomy (partial, total, etc.) performed varied depending on the number of polyps present^24^. Our study, along with others, shows that the number of polyps in MUTYH-mutated patients typically ranges from 10 to 100^9,18^. In line with prior research studies, most of our cases also exhibited colorectal polyposis^18,24^. We have evaluated homozygous and heterozygous MUTYH mutations. Therefore, the proportion of cases with polyposis in our cohort was lower than previously reported. Only one homozygous case presented without polyposis in our cohort.

As compatible with literature data, MUTYH-mutated CRC was seen at an average age of 48 years^15,18,24^. Although it has not been addressed explicitly in the existing literature, our research has revealed a notable male predominance. This observation may be attributed to the fact that colorectal cancer is more prevalent in males^1^. Surgery was the most common first-line treatment in MAP; and similar to our study, most cases in the literature had reportedly undergone total colectomy.

Our findings that align with those of previous studies revealed that MUTYH-mutated CRC is predominantly located in the proximal colon. Metachronous and synchronous CRCs are known occurrences in patients with MUTYH mutations^16,18,24^. In our study, four cases of synchronous CRCs were identified. Besides, average tumor diameter in our MUTYH-mutated CRC cases was 4.3 cm. Interestingly, pT3 was the most common pathological T stage detected in our study. In the study of Castillejo et al., three out of seven homozygous cases with MUTYH mutations had a pT3 CRC^16^. Consistent with other studies, most of the CRCs in our cohort were moderately differentiated^24^. A novel finding of our study was the presence of lymph node metastases in 45.5% of cases including one pN1 and four pN2 cases.

Concomitant MMR deficiency and MUTYH mutation is a rare occurrence, only briefly discussed in the literature^15-17,29^. In our cohort, one case with a heterozygous and one case with a homozygous MUTYH mutation were MSH2-deficient. In the case of a heterozygous MUTYH mutation, CRC localized in the ascending colon exhibited loss of MSH2 and MSH6 proteins. In the study of Castillejo et al., seven out of 225 cases with Lynch-like syndrome had a homozygous MUTYH mutation, and eight of them carried a heterozygous MUTYH mutation. Three cases with homozygous MUTYH mutations were deficient for MLH1 and PMS2 proteins. Indicated number of cases with heterozygous MUTYH mutations were MLH1 and PMS2-deficient (n=3), MSH2-deficient (n=1), isolated MSH6-deficient (n=1), and isolated PMS2-deficient (n=1)^16^. In the cohort of dos Santos et al., the CRC of one MUTYH-mutated patient demonstrated loss of MLH1, PMS2, and MSH6 proteins^17^. In the study of Giráldez et al., one CRC case out of eight MUTYH mutation carriers was MSH6-deficient^15^. As data is gathered, the incidence of such cases may expectedly increase due to the overlap in DNA base excision repair pathways^17,29^. All of four cases with a c.800C>T p.P267L mutations had an intact MMR protein. Given the limited number of cases, it is challenging to draw conclusions about the specific mutations in MUTYH.

The fact that the study was conducted with a limited number of cases was the main limitation of our study. It is hard to make universally applicable conclusions based on our limited data. Besides, this study did not contain a control group that did not carry a MUTYH mutation with which the group of patients with a MUTYH mutation would be compared. A recent paper on early onset CRC in the hospital where our study was conducted has been published. This cohort consisted of colorectal cancer patients ≤50 years old. Out of 130 cases, 30 cases (22.9%) were MMR-deficient, while 9.2% (n=12) of these cases were MSH2- deficient^30^. Our cohort of MSH2-deficient cases consisted of a heterogeneous age group with ages of the patients varying between 40 and 58 years. Therefore, it is hard to compare our data with the findings of this aforementioned study.

## CONCLUSION

This study provides the first clinicopathological characterization of MUTYH-mutated colorectal cancer patients in the Turkish population. It is also the first case report on tumor diameter and lymph node status in MUTYH-mutated colorectal cancers. We also report two illustrative cases that highlighted the association between MUTYH mutation and MMR-deficiency. Herein, a case of polyposis associated with colorectal cancer and a double heterozygous MUTYH mutation was presented. In future studies, broader cohorts comprising cases with MUTYH-mutated and non-mutated colorectal cancers should be investigated to further improve treatment options for these patients. Older age, undergoing incomplete partial colectomies or undersampling of the resected colon segment can cause underdiagnosis of MAP. Therefore, family history of colorectal cancer and sequencing for rare variants of MUTYH mutations can help diagnose MUTYH-associated polyposis cases.

## Data Availability

The datasets generated and/or analyzed during the current study are available from the corresponding author upon reasonable request.
